# Pre- and intra-operative predictors of postoperative hospital length of stay in patients undergoing radical prostatectomy for prostate cancer in China: a retrospective observational study

**DOI:** 10.1186/s12894-018-0351-6

**Published:** 2018-05-18

**Authors:** Qingmei Huang, Ping Jiang, Lina Feng, Liping Xie, Shuo Wang, Dan Xia, Baihua Shen, Baiye Jin, Li Zheng, Wei Wang

**Affiliations:** Department of Urology, The First Affiliated Hospital, College of Medicine, Zhejiang University, 79 Qingchun Road, Hangzhou, 310003 Zhejiang China

**Keywords:** Prostate cancer, Radical prostatectomy, Predictor, Length of stay, China

## Abstract

**Background:**

Hospital length of stay (LOS) has recently been receiving increasing attention as a marker of medical resource consumption. Identifying predictors of longer LOS can better equip doctors to counsel patients and facilitate more efficient patient flow and utilization of medical resources. The objective of this study was to identify pre- and intra-operative risk factors for postoperative hospital LOS in patients who had undergone radical prostatectomy in China.

**Methods:**

We retrospectively analyzed data of 793 eligible patients with prostate cancer who had undergone radical prostatectomy in our institution between January 2011 and March 2016. Relevant preoperative variables, including patient characteristics, medical comorbidities, prostate cancer disease-specific variables, urinary tract symptoms, preoperative laboratory values, and intraoperative variables including operation type, operation duration, and blood loss, were analyzed. The outcome was postoperative length of stay which was calculated as the time from the date of operation to the date of discharge. Multiple linear regression analysis was used to identify predictors of this outcome.

**Results:**

The mean postoperative LOS was 11.7 days (±4.6 days) and the median 10 days (range, 5–46 days). According to univariate and multivariate analysis, operation type (open or laparoscopic), blood loss, Gleason score (≥8) and preoperative laboratory values of white blood count (WBC) were found to be the main explanatory predictors of postoperative LOS of patients with prostate cancer in our institution. Additionally, open surgery was the strongest significant predictor of longer LOS according to the standardized coefficients in this model.

**Conclusions:**

Our findings indicate that significant predictors of longer postoperative LOS in patients who have undergone radical prostatectomy in China include both preoperative variables of Gleason score, WBC and intraoperative variables of operation type (open or laparoscopic), blood loss. To shorten hospital LOS in patients with prostate cancer and optimize utilization of Chinese medical resources, efforts should be made to improve the intraoperative process and reduce the prevalence of preoperative risk factors.

## Background

Prostate cancer is the commonest cancer in men [1] and radical prostatectomy is one of the main treatments for clinically localized prostate cancer [2]. With increasing aging of China’s population, the number of patients with newly diagnosed prostate cancer has been increasing continuously in recent years [3, 4], challenging Chinese medical institutions to provide adequate care despite limited medical resources. In recent years, hospital length of stay (LOS) has been increasingly used as a marker for medical resource consumption [5–7]. Prolonged LOS is not only associated with higher medical costs and resource consumption [5, 8], but may also place patients at greater risk of complications, including hospital-acquired infections and deep vein thrombosis [9, 10]. In China, there is another important consideration regarding LOS, especially for patients who are to undergo elective surgery, including radical prostatectomy. The limited number of hospital beds means that such patients must wait for a bed to become available, which frequently depends on other patients being discharged. Thus, it is important to identify risk factors for prolonged LOS and provide strategies for shortening LOS and reducing unnecessary resource utilization.

Numerous risk factors are associated with prolonged LOS, including preoperative and intraoperative factors and postoperative complications [11]. Studies focusing on preoperative risk factors have pointed out that some of them are important predictors of LOS [12]. One recent study evaluating factors that predict longer hospital stay in patients who have undergone robot-assisted radical prostatectomy (RARP) identified patient comorbidity as the only independent preoperative predictor of prolonged hospital LOS [13]. Previous studies exploring both pre- and intra-operative risk factors for prolonged LOS after commonly-performed urologic surgery, including prostatectomy, have identified some with significant impact, including older age, low hematocrit, high creatinine, operation duration, and intraoperative transfusion [11, 14]. However, because these researchers did not analyze disease-specific variables, these factors remain unexplored for patients with prostate cancer.

Elucidating risk factors that are significantly associated with LOS may help physicians to identify patients at greater risk for prolonged LOS and thus provide more appropriate counseling [12], as well as ultimately facilitating more efficient patient flow and operations management. However, the findings of studies conducted in Western countries may not be applicable to Chinese men with prostate cancer [15]. As far as we know, no studies have explored risk factors related to prolonged hospital LOS in Chinese inpatients who have undergone radical prostatectomy for prostate cancer. We therefore comprehensively collected possible risk factors, including patient characteristics, comorbidities, disease-specific variables, urinary tract symptoms, preoperative laboratory values, and intraoperative variables, with the aim of examining pre- and intra-operative predictors of prolonged LOS for prostate cancer patients in China.

## Methods

### Study sample

**Fig. 1:**
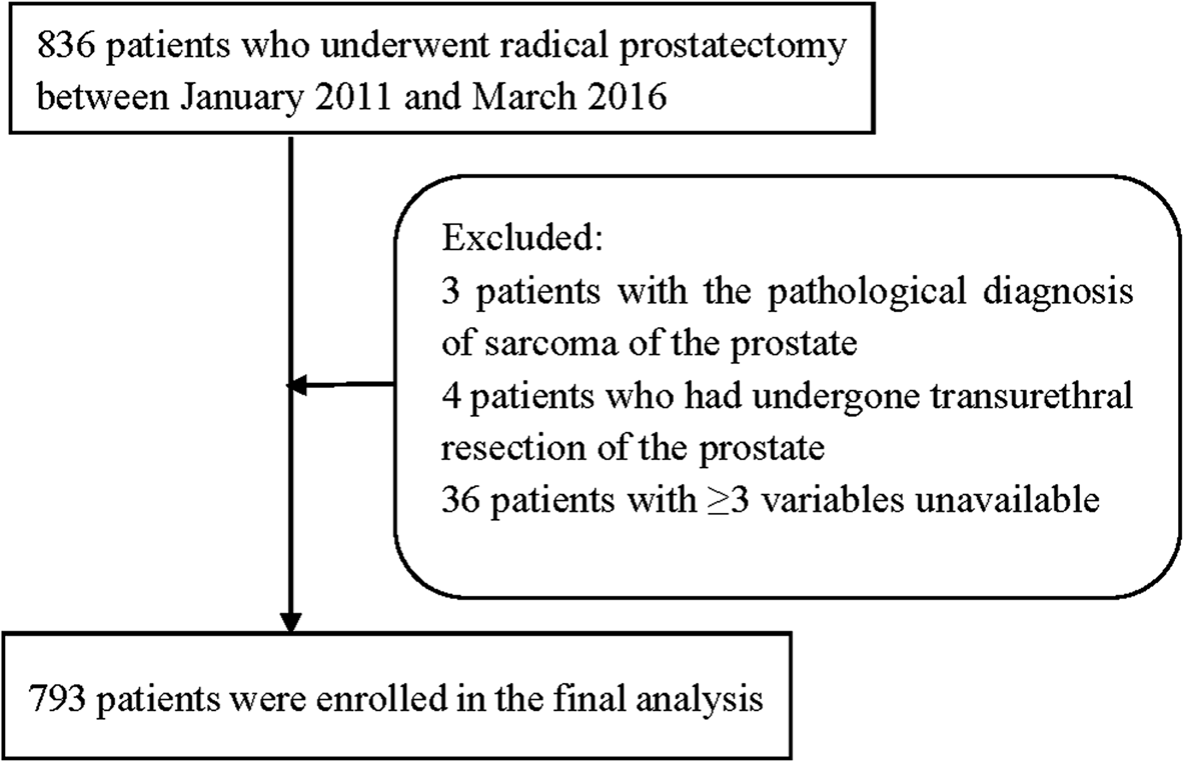
Flowchart of study cohort screening

Between January 2011 and March 2016, 836 consecutive patients with localized prostate cancer underwent radical prostatectomy and were discharged from our institution. Only patients with the pathological diagnosis of prostatic adenocarcinoma were included in this study, those with sarcoma of the prostate being excluded. Patients who had undergone transurethral resection of the prostate or another operation for concomitant diseases during the period of hospitalization were also excluded. Additionally, patients for whom equal to or more than three study variables were unavailable were also excluded from the final analysis. All patients’ data were extracted by a trained clinical reviewer from electronic medical records maintained in a secure clinical database at our institution. After applying inclusion and exclusion criteria, 793 inpatients were included in the final analysis. The flowchart of screening for eligibility for the study is shown in Fig. 1.

### Dependent variable: Postoperative LOS

The primary outcome variable was postoperative LOS in days, which was calculated from the date of operation to the date of discharge. Because the data of LOS in days were not normally distributed, these data were subjected to reciprocal transformation to meet the model assumption of a normal distribution. The reciprocally transformed LOS was then used as the dependent variable in the subsequent multiple linear regression analysis.

### Independent variables measured

In this study, 32 variables were assessed. Preoperative variables included patient characteristics, comorbidities, prostate cancer disease-specific variables, urinary tract symptoms, and preoperative laboratory values. Patient characteristics assessed included age, body mass index (BMI), marital status, smoking and drinking status, and family history of prostate cancer. Comorbidities, including hypertension requiring medication; diabetes requiring either oral medication or insulin injections or both; and history of cardiovascular and cerebrovascular disease were combined into one variable because of the small positive sample size. History of cardiovascular and cerebrovascular disease was treated as a dichotomous variable and defined as positive in the presence of a history of myocardial infarction, coronary artery disease before or after coronary stent implantation, arrhythmia, transient ischemic attack, or cerebrovascular accident such as cerebral embolism, cerebral thrombosis, or cerebral hemorrhage. The above information was abstracted from preoperative medical records. Inclusion of the disease-specific variable of prostate specific antigen (PSA) was considered essential; the most recent PSA value prior to surgery was used in the analysis. Biopsy Gleason scores were acquired from prostate biopsy reports. Prostate volume was calculated by using the following formula: transverse diameter × vertical diameter × longitudinal diameter × π/6; the diameters were measured by color ultrasound. Urinary tract symptoms, including dysuria, pain or burning on urination, frequent urination, urgent urination, and hematuria, were assessed as dichotomous variables and abstracted from medical records of symptoms reported by patients. Preoperative laboratory values closest to the day of surgery were also recorded, including white blood count (× 10^9^/L) (WBC), neutrophil count (%), hemoglobin, hematocrit, platelet count (× 10^9^/L), albumin, glutamic-pyruvic transaminase (GPT), serum total bilirubin, creatinine, serum potassium, serum calcium, and blood glucose. Intraoperative variables, including operation duration (h), blood loss (L), and operation type were collected from operation notes and operation logs. The type of surgery performed (open radical prostatectomy [ORP], laparoscopic radical prostatectomy [LRP], or robot-assisted radical prostatectomy [RARP]) was selected by each patient after discussion with the surgeon.

### Statistical analysis

Continuous data are presented as mean ± standard deviation or median (interquartile range) and categorical data as frequency and percentage. Correlations between each variable and LOS were tested by univariate analysis, including the Mann–Whitney U test for variables with two subgroups, Kruskal–Wallis test for variables with multiple subgroups and Spearman correlation analysis for continuous variables. Variables that were found to be significantly associated with LOS (*p* ≤ 0.1; two-sided probability) in the univariate analysis were included in the subsequent multiple linear regression analysis by using stepwise selection methods. *P* < 0.05 was set as the criterion for inclusion of a variable in the final model. There were missing data in the variables of BMI, Prostate volume and Operation duration, while the extent of cases with one or two missing variables in the 793 patients was less than 3% and there were no statistically difference in LOS between cases with versus without missing data. Missing data were imputed by mean substitution and were included in the data analysis. All statistical analyses were performed using SPSS software (version 17.0, SPSS).

## Results

**Fig. 2:**
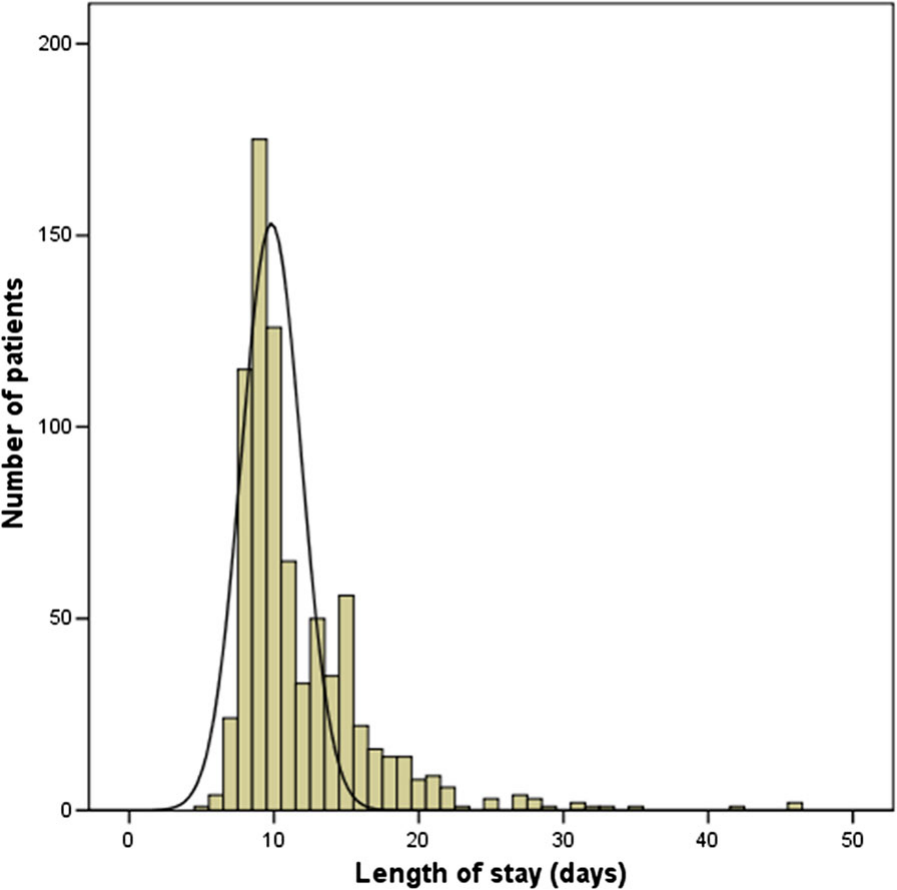
Histogram illustrating the distribution of postoperative length of stay for all patients in this sample

The mean postoperative LOS for the entire sample was 11.7 days (± 4.6 days), and the median 10 days (range: 5–46 days; Fig. 2). The mean age of the 793 study patients was 67.0 ± 6.8 years and the mean BMI 23.7 ± 2.8 kg/m^2^. The mean preoperative PSA value was 17.0 ± 22.3 ng/mL (range: 0.009–363.3 ng/mL), with 191 patients’ (24.1%) value being in the high-risk range (PSA > 20 ng/mL). As to type of surgery, 325 patients (41.0%) had undergone ORP, 257 (32.4%) LRP, and 211 (26.6%) RARP. The mean operation time (hours) was 3.0 ± 0.9 h. Details of patient characteristics and pre- and intra-operative variables are listed in Table 1.

**Table 1:** Characteristics of the 793 patients and variables associated with LOS by univariate analysis

Variables	Mean ± SD or n (%)	LOS		*P* value
		Mean ± SD	Median (25th–75th percentile)	
Patient demographics				
Age (y)	67.0 ± 6.8	11.7 ± 4.6	10.0 (9.0–14.0)	0.693^a^
Body mass index (kg/m^2^)	23.7 ± 2.8	11.7 ± 4.6	10.0 (9.0–14.0)	0.407^a^
Marital status				0.877^c^
Married	771 (97.2%)	11.7 ± 4.6	10.0 (9.0–14.0)	
Widowed	16 (2.0%)	11.8 ± 6.1	10.0 (9.0–11.8)	
Single	6 (0.8%)	11.8 ± 3.7	10.0 (9.0–16.3)	
Smoking				0.612^b^
No	511 (64.4%)	11.8 ± 4.5	10.0 (9.0–14.0)	
Yes	282 (35.6%)	11.6 ± 4.8	10.0 (9.0–13.3)	
Drinking				0.678^b^
No	621 (78.3%)	11.7 ± 4.5	10.0 (9.0–13.5)	
Yes	172 (21.7%)	11.7 ± 5.2	10.0 (9.0–14.0)	
Family history of Prostate cancer				0.752^b^
No	580 (73.1%)	11.7 ± 4.8	10.0 (9.0–14.0)	
Yes	213 (26.9%)	11.7 ± 4.3	10.0 (9.0–13.0)	
Medical comorbidities				
Hypertension				0.798^b^
No	434 (54.7%)	11.8 ± 5.1	10.0 (9.0–13.3)	
Yes	359 (45.3%)	11.6 ± 4.1	10.0 (9.0–14.0)	
Diabetes				0.487^b^
No	718 (90.5%)	11.8 ± 4.8	10.0 (9.0–13.0)	
Yes	75 (9.5%)	11.3 ± 3.5	10.0 (8.0–14.0)	
Cardiovascular & cerebrovascular diseases				0.705^b^
No	734 (92.6%)	11.7 ± 4.6	10.0 (9.0–13.0)	
Yes	59 (7.4%)	12.0 ± 5.0	10.0 (9.0–15.0)	
Prostate cancer disease-specific variables				
Biopsy Gleason score				**< 0.001** ^**c**^
≤ 6	254 (32.0%)	11.1 ± 4.2	10.0 (9.0–12.0)	
7	344 (43.4%)	11.7 ± 5.0	10.0 (9.0–13.0)	
≥ 8	195 (24.6%)	12.6 ± 4.5	11.0 (9.0–15.0)	
Preoperative PSA	17.0 ± 22.3	11.7 ± 4.6	10.0 (9.0–14.0)	**0.036** ^a^
Prostate Volume (ml)	34.0 ± 19.6	11.7 ± 4.6	10.0 (9.0–14.0)	0.214^a^
Urinary tract problems				
Dysuresia				**0.032** ^b^
No	667 (84.1%)	11.6 ± 4.7	10.0 (9.0–13.0)	
Yes	126 (15.9%)	12.3 ± 4.3	11.0 (9.0–15.0)	
Pain or burning on urination				0.416^b^
No	761 (96.0%)	11.7 ± 4.7	10.0 (9.0–13.0)	
Yes	32 (4.0%)	11.9 ± 3.6	10.5 (9.0–15.0)	
Frequent urination				0.169^b^
No	633 (79.8%)	11.7 ± 4.9	10.0 (9.0–13.0)	
Yes	160 (20.2%)	11.8 ± 3.7	10.0 (9.0–14.0)	
Urgent urination				0.912^b^
No	690 (87.0%)	11.8 ± 4.8	10.0 (9.0–14.0)	
Yes	103 (13.0%)	11.4 ± 3.6	10.0 (9.0–14.0)	
Hematuria				0.545^b^
No	776 (97.9%)	11.7 ± 4.7	10.0 (9.0–14.0)	
Yes	17 (2.1%)	11.1 ± 3.8	10.0 (8.0–12.5)	
Preoperative laboratory values				
White blood count (×10^9^/L)	5.8 ± 1.5	11.7 ± 4.6	10.0 (9.0–14.0)	**< 0.001** ^a^
Neutrophil (%)	57.0 ± 8.9	11.7 ± 4.6	10.0 (9.0–14.0)	0.467^a^
Hemoglobin (g/L)	143.3 ± 13.4	11.7 ± 4.6	10.0 (9.0–14.0)	0.713^a^
Hematocrit (%)	42.1 ± 3.6	11.7 ± 4.6	10.0 (9.0–14.0)	0.387^a^
Platelet count (× 10^9^/L)	186.9 ± 49.3	11.7 ± 4.6	10.0 (9.0–14.0)	**0.005** ^a^
Albumin (g/L)	43.2 ± 4.2	11.7 ± 4.6	10.0 (9.0–14.0)	0.475^a^
GPT (U/L)	23.6 ± 21.2	11.7 ± 4.6	10.0 (9.0–14.0)	0.170^a^
Serum total bilirubin (ummol/L)	12.3 ± 5.0	11.7 ± 4.6	10.0 (9.0–14.0)	0.402^a^
Creatinine (ummol/L)	78.4 ± 13.2	11.7 ± 4.6	10.0 (9.0–14.0)	0.106^a^
Serum potassium (mmol/L)	4.2 ± 0.4	11.7 ± 4.6	10.0 (9.0–14.0)	0.309^a^
Serum calcium (mmol/L)	2.3 ± 0.1	11.7 ± 4.6	10.0 (9.0–14.0)	**0.008** ^a^
Blood glucose (mmol/L)	5.0 ± 0.9	11.7 ± 4.6	10.0 (9.0–14.0)	**0.053** ^a^
Intraoperative variables				
Operation type^&^				**< 0.001** ^c^
Open	325 (41.0%)	13.3 ± 5.1	12.0 (10.0–15.0)	
Laparoscopic	257 (32.4%	11.0 ± 3.8	10.0 (9.0–12.0)	
Robot assisted	211 (26.6%)	10.1 ± 4.1	9.0 (8.0–10.0)	
Blood loss (L)	0.2 ± 0.2	11.7 ± 4.6	10.0 (9.0–14.0)	**< 0.001** ^a^
Operation duration (h)	3.0 ± 0.9	11.7 ± 4.6	10.0 (9.0–14.0)	0.177^a^

SD, standard deviation; LOS, length of stay; PSA, prostate specific antigen; GPT, Glutamic-pyruvic transaminase

Bold values indicate significant *p* values (*p* ≤ 0.1)

^&^A LSD post hoc test revealed that all three subgroups were significantly different from each other

^a^Spearman correlation analysis

^b^Mann-Whitney *U* test

^c^Kruskal-Wallis H(K) test

Table 1 shows variables significantly associated with LOS in days according to univariate analysis (*P* value ≤0.1). Both some pre- and intra-operative variables were associated with longer postoperative LOS; namely, the prostate cancer disease-specific variables of preoperative PSA value and biopsy Gleason score, urinary tract symptoms of dysuria, preoperative laboratory values for WBC, platelet count, serum calcium and blood glucose, and intraoperative variables of operation type, and blood loss. However, patient age was not a risk factor for longer LOS. Additionally, the intraoperative variable of operation duration was not found to be significantly associated with longer postoperative LOS. The same results were obtained when reciprocal-transformed LOS was subjected to univariate analysis.

Table 2 summarizes the results of multiple linear regression analysis. The intraoperative variables of operation type (open or laparoscopic) and blood loss, the disease-specific variable of Gleason score, and preoperative laboratory values of WBC were found to be the main explanatory factors for postoperative LOS of prostate cancer patients in our institution. Of the above variables, the operation type of open was the strongest significant predictor for longer LOS (β = − 0.325), followed by blood loss (β = − 0.205) according to the standardized coefficients (negative coefficients corresponding to longer LOS because of the reciprocal transformation). In addition, the disease-specific variable of Gleason score was an important predictor of longer postoperative LOS. The variable of WBC was inversely associated with LOS, which means that a lower preoperative white blood count is associated with a longer postoperative LOS. The R-squared value (indicator of fit) for the entire model is 20.5%, indicating that all of the significant variables in the model account for 20.5% variance in the postoperative LOS of men undergoing surgery for prostate cancer in China. In order to easily interpret the effect of the unstandardized coefficients of the significant factors on LOS in days, we take the 50th percentile of LOS (10 days) for example. Specifically, if the open operation was conducted, the length of hospital stay would be prolonged to be 12.05 days, increased about 2 days compared with operation of RARP. While the operation of laparoscopic would increase LOS by 0.87 day or in other words more than a half day but less than 1 day. Additionally, 1 unit (L) greater blood loss would prolong the 50th percentile of LOS to be 12.99 days, increased by about 3 days and Gleason score ≥ 8 would increase LOS by about 1 day compared to the reference category of Gleason score ≤ 6. However, for the effect of WBC, one unit decrease in WBC would slightly increase the 50th percentile of LOS by 0.2 day.

**Table 2:** The results of multiple linear regression analysis

Variables	Unstandardized coefficient^a^	Standardized coefficient (*β*)^a^	t value	*P* value
Operation type				
Robot assisted	REF	–	–	–
Open	−0.017	−0.325	−7.259	**< 0.001**
Laparoscopic	−0.008	−0.146	−3.730	**< 0.001**
Blood loss (L)	−0.023	−0.205	−5.438	**< 0.001**
Gleason score				
≤ 6	REF	–	–	–
7	−0.003	−0.061	− 1.668	0.096
≥ 8	−0.009	−0.152	−4.115	**< 0.001**
White blood count (× 10^9^/L)	0.002	0.089	2.775	**0.006**
Preoperative PSA	8.222E-6	0.007	0.208	0.835
Dysuresia	−0.002	−0.027	−0.826	0.409
Platelet count (× 10^9^/L)	2.816E-5	0.053	1.550	0.122
Serum calcium (mmol/L)	−0.012	− 0.058	−1.797	0.073
Blood glucose (mmol/L)	0.001	0.043	1.352	0.177

REF, referent; —, not applicable

Bold items indicate factors significantly associated with LOS (*p* values < 0.05)

^a^negative coefficients corresponding to longer LOS because of the reciprocal transformation

## Discussion

The expected increasing numbers of patients with prostate cancer will inevitably increase the demand for access to hospitalization; it is therefore important to optimize medical resource utilization in an attempt to meet this demand. Hospital LOS, an important indicator of resource utilization, has been increasingly investigated in the face of limited medical resources and increasing pressure for cost containment [5, 7, 15]. Identifying risk factors that influence LOS of patients with prostate cancer in China will enable more accurate prediction of bed flow and access and thus facilitate more efficient resource allocation. In our study, we found that the preoperative variables of biopsy Gleason score (≥ 8) and WBC and intraoperative variables of operation type (open or laparoscopic), and blood loss are significant predictors of longer LOS for post-radical prostatectomy patients with prostate cancer in China.

In our study, the median postoperative LOS of our patient cohort was 10 days, which is much longer than that reported from Western countries, where the median postoperative LOS after radical prostatectomy is reportedly only one day [13, 14]. This discrepancy is likely attributable to the huge differences in healthcare systems, medical insurance status, admission/discharge policies, and socio-cultural factors between China and these countries [16]. First, in China, the lack of post-hospitalization care such as that provided by rehabilitation centers and clinician’s follow-up checks, which are commonly available in Western countries [15, 17], lengthens the LOS. In China, most post-radical prostatectomy patients stay in hospital until they have achieved stable physical fitness. For example, the discharge criteria in our institution include normal vital signs, return of bowel function, ambulation without assistance, and removal of pelvic drainage tubes, unlike the criteria in Western hospitals [13]. What’s more, the structure of healthcare financing may also contribute to the longer LOS in China than in Western countries. For example, the USA Medicare prospective payment system and diagnosis-related group-based payment system for hospitalization provide a financial incentive for earlier hospital discharge [18], whereas China’s healthcare system generally provides treatments and care independent of LOS or costs [16, 19]. Indeed, the cost per night (bed expense: about $4.43) is much lower in our hospitals than in Western countries [20]. Traditionally, many patients are willing to stay longer in hospital, where medical treatment and nursing care are easily accessible, to ensure they are in good physical condition before discharge rather than agreeing to earlier discharge with the attendant risks of developing complications outside hospital. Another noteworthy point is that Chinese doctors tend to be more conservative and cautious in ensuring that patients achieve a stable state before discharging them from hospital, their motivation being to avoid potential challenges, legal action, or even threats from patients or their families, as have occurred in association with tense doctor–patient relationships in China, especially in recent years [21, 22]. All the above reasons likely contribute to the much longer LOS for both patients with prostate cancer and those with other diseases [15, 17, 23] in China than in Western studies.

Being one of the main treatments for clinically localized prostate cancer, three types of radical prostatectomy are available in our institution, namely open, laparoscopic, and robotic prostatectomy. Many previous studies comparing operative outcomes of the three types of surgery, have reported that minimally invasive (laparoscopic or robotic) surgery is associated with a significantly shorter LOS than open surgery [24–28]. Our findings were consistent with this in that patients undergoing open radical prostatectomy had longer LOS than both patients undergoing LRP and RARP. In fact, undergoing open radical prostatectomy was the strongest predictor of LOS according to multivariate analysis in our study. It is noteworthy that operation type of laparoscopic was also a significant predictor of LOS by multivariate analysis and a post hoc comparison between the three operation types revealed a significantly longer LOS for patients undergoing LRP than RARP on univariate analysis. Previous studies have highlighted the advantages of RARP over ORP [24–28] and LRP [29, 30]; specifically, less blood loss, lower transfusion rate, lower complication rates, and better functional outcomes [24–30]. Shorter hospital stay of RARP over ORP have also been widely reported [24–28] while our study identified that not only for ORP but also for LRP, RARP has shorter LOS, which differs from reports from Western countries [29]. Relevant point to this discrepancy is that a large proportion of patents in our study had undergone ORP (41.0%) or LRP (32.4%), only 26.6% patients having undergone RARP, unlike in Western countries, where much greater proportion of patients reportedly undergo RARP [13, 14, 31]. This may be another explanation for postoperative LOS being so much longer for patients with prostate cancer in China than for those in Western countries. Although RARP has many advantages over LRP and ORP [24–30], it is much more costly than the other two options [20, 27]. Therefore, RARP may be the optimal choice for affluent patients.

We also found the intraoperative variable of blood loss to be an important predictor of longer LOS after radical prostatectomy. This result is consistent with previous studies [11, 14] that analyzed data from the National Surgery Quality Improvement Program database to explore the risk factors for prolonged LOS after commonly performed urologic surgical procedures, including nephrectomy and prostatectomy, and concluded that intraoperative transfusion is significantly associated with longer postoperative LOS. In addition, the above studies [11, 14] and other related studies [31–33] were consistent in finding that the intraoperative variable of operation duration is also significantly associated with longer LOS. However, our findings were inconsistent with these in that operation duration was not a significant predictor of longer LOS. In this context, it is noteworthy that one recent study exploring risk factors for hospital LOS in patients undergoing RARP drew a contradictory conclusion [13], reporting a shorter operative time with prolonged hospital LOS on univariate analysis but lack of support for this finding by multivariate analysis, indicating that the variable of operation duration as predictor of LOS after radical prostatectomy may need further investigation.

In one previous study [13], the disease-specific variable of Gleason score was also evaluated but not found to be significant, which conflicts with our findings. In our study, we abstracted and analyzed the prostate cancer disease-specific variables of preoperative PSA, biopsy Gleason score, and prostate volume and found that preoperative PSA and biopsy Gleason score were both associated with LOS by univariate analysis. However, only Gleason score ≥ 8 was an important predictor of longer LOS by multivariate analysis. Patients with higher biopsy Gleason scores (≥ 8) may thus be at risk of longer postoperative LOS after radical prostatectomy. To the best of our knowledge, this is the first report of a significant association between this variable and hospital LOS in post-prostatectomy patients. However, there are few studies on the impact of prostate cancer disease-specific risk factors on hospital LOS. Further prospective research is needed to validate the significance of Gleason score as a predictor of prolonged LOS.

Studies investigating predictors of LOS in other surgical disciplines have also highlighted that preoperative laboratory values such as low hematocrit, high creatinine, or low albumin are significant indicators of prolonged LOS [12, 14, 34]. We also abstracted these preoperative laboratory values, but found that hematocrit, creatinine or albumin were not significantly associated with LOS in our patient cohort, whereas white blood count, platelet count, serum calcium and blood glucose were. However, of these variables, only white blood count persisted into the final model after multiple linear regression analysis. It makes intuitive sense that higher white blood counts would indicate tissue infection and inflammation that would likely lengthen hospital stay. However, in our study the results were contradictory in that WBC was inversely associated with LOS, meaning that lower preoperative WBC were associated with longer postoperative LOS. To better understand this puzzling result, we made a further analysis by categorizing the variable of WBC as abnormal low, normal, abnormal high on the basis of clinical cutoff points. And results showed that the percent of patients with abnormal low WBC was six-fold greater than the percent of patients with abnormal high WBC and a post hoc comparison revealed that patients with abnormal low WBC had significant longer LOS than patients with normal WBC while no statistically significant difference exist between patients with abnormal high WBC and patients with normal WBC. This confusing result may indicate that prostate cancer patients may be more easily in an underling condition. Because the lower WBC may be an indication of that patients were with severe infections, or radiotherapy/chemotherapy or other unfitness physical state [35, 36], which may result in longer postoperative LOS. However, given the limitations of few studies exploring the relationship between this variable and LOS in prostate cancer patients, more studies are needed to further verify or refute this result of our study.

We here identified several important predictors of longer LOS for post-prostatectomy patients with prostate cancer in China. In particular, we added the disease-specific variable of Gleason score as a risk factor for longer LOS. However, several limitations of our study should be noted. First, it was a retrospective analysis and data were collected from a single institution in Zhejiang, China, thus limiting the generalizability of these finding to other countries or even other institutions in China. Second, we only analyzed preoperative and intraoperative variables and the R^2^ value in the final regression model was 0.205; such a small R^2^ value suggests that postoperative variables such as complications may play major roles in postoperative LOS. Previous studies have reported strong associations between postoperative adverse events and prolonged LOS [11]. Hence, future analyses should include postoperative variables to enable more comprehensive exploration of risk factors that impact LOS after radical prostatectomy in China. Third, the radical prostatectomies of the 793 patients analyzed were performed by different surgeons in our institution. Because we could not quantify the levels of skill of the surgeons involved, we were unable to draw conclusions about their influence on LOS. Last but not least, because countries differ greatly in their healthcare policies, our results should be considered in the context of country-specific healthcare systems and medical–cultural environments when comparing them with results from other countries. Additionally, the influence of doctors’ and patients’ attitudes to LOS should not be ignored because the doctor–patient relationship tends to be more strained in China than in Western countries.

## Conclusions

Elucidating the risk factors for longer LOS will enable better patient counseling and more efficient management of medical resources. In our study, we found that both some preoperative and intraoperative variables are significant predictors of longer postoperative LOS after radical prostatectomy in China. Measures should be taken to improve intraoperative procedures and reduce the prevalence of preoperative risk factors without sacrificing the quality of medical care with the aim of shortening hospital LOS and improving the efficiency of utilization of Chinese medical resources.

## Abbreviations

BMI: Body mass index
GPT: Glutamic-pyruvic transaminase
LOS: Length of stay
LRP: Laparoscopic radical prostatectomy
ORP: Open radical prostatectomy
PSA: Prostate specific antigen
RARP: Robot-assisted radical prostatectomy
REF: Referent
SD: Standard deviation
SE: Standard error
WBC: White blood count
